# Towards inoculant development for Bambara groundnut (*Vigna subterranean* (L.) Verdc) pulse crop production in Namibia

**DOI:** 10.3389/fpls.2023.1270356

**Published:** 2023-10-26

**Authors:** Abhijit Sarkar, Felicitas M. Fwanyanga, Lydia N. Horn, Sina Welzel, Marco Diederichs, Luca Jonas Kerk, Meret Zimmermann, Barbara Reinhold-Hurek

**Affiliations:** ^1^ CBIB Center for Biomolecular Interactions Bremen, Department of Microbe-Plant Interactions, Faculty of Biology and Chemistry, University of Bremen, Bremen, Germany; ^2^ Zero Emissions Research Initiative, Multi-disciplinary Research Services, University of Namibia, Windhoek, Namibia

**Keywords:** Bambara groundnut, legumes, *Bradyrhizobium*, bioinoculant, Kavango

## Abstract

**Introduction:**

The globally expanding population, together with climate change, poses a risk to the availability of food for humankind. Bambara groundnut (BGN) (*Vigna subterranea* (L.) Verdc) is a neglected, relatively drought-tolerant native legume of Sub-Saharan Africa that has the potential to become a successful food crop because of its nutritional quality and climate-smart features. Nitrogen fixation from root nodule symbiosis with climate-adapted rhizobial symbionts can contribute nitrogen and organic material in nutrient-poor soil and improve yields. However, high soil temperature and drought often reduce the abundance of native rhizobia in such soil. Therefore, the formulation of climate-smart biofertilizers has the potential to improve the farming of BGN at a low cost in a sustainable manner.

**Method:**

The effect of seven *Bradyrhizobium* spp. strains native to Namibia, including *B. vignae* and *B. subterraneum*, were tested on three Namibian BGN varieties (red, brown, cream) in greenhouse pot experiments in Namibia, using soil from the target region of Kavango. Each variety was treated with a mixed inoculant consisting of seven preselected strains (“MK”) as well as with one promising single inoculant strain.

**Results:**

The results revealed that in all three varieties, the two inoculants (mixed or single) outperformed the non-inoculated cultivars in terms of shoot dry weight by up to 70%; the mixed inoculant treatment performed significantly better (p < 0.05) in all cases compared to the single inoculant used. To test whether the inoculant strains were established in root nodules, they were identified by sequence analysis. In many cases, the indigenous strains of Kavango soil outcompeted the inoculant strains of the mix for nodule occupancy, depending on the BGN variety. As a further preselection, each of the individual strains of the mix was used to inoculate the three varieties under sterile conditions in a phytotron. The agronomic trait and root nodulation response of the host plant inoculations strongly differed with the BGN variety. Even competitiveness in nodule occupancy without involving any indigenous strains from soil differed and depended strictly on the variety.

**Discussion:**

Severe differences in symbiont-plant interactions appear to occur in BGN depending on the plant variety, demanding for coupling of breeding efforts with selecting efficient inoculant strains.

## 1 Introduction

While several agricultural research projects have typically concentrated on staple crops, scientists in industrialized nations have paid less attention to underutilized and neglected crop species (Ajayi et al., 2020). One example of an underutilized crop is the Bambara groundnut (*Vigna subterranea* (L.) Verdc), a legume crop grown for human consumption in many parts of the world, particularly in Africa (Guei et al., 2019). The Bambara groundnut (BGN), like other legumes, can fix nitrogen through root nodule symbiosis (Hillocks et al., 2012). This feature gives legumes an advantage over other plant species in N-limited soils because they can convert atmospheric N_2_ into forms that plants can use through their symbiotic relationship, with rhizobia fulfilling complete or partial nitrogen requirements of the legume (Allito et al., 2021; Bünger et al., 2021). It can also help to build up nitrogen in soils that can be used by succeeding non-legume crops (Mohammed et al., 2021).

Despite its importance, the production of BGN is hindered by poor crop establishment, improper planting depth and plant spacing, the use of unimproved seeds, insufficient soil fertility, and a lack of efficient nodulation technology (Ikenganyia et al., 2018). For instance, it has been reported that local farmers in Namibia have experienced low yields and declining soil nutrient levels, with low natural nodulation rates in pulses like BGN (Grönemeyer et al., 2014). Additionally, water stress can also be a limitation for the nodulation of cowpeas and, in combination with soil type, can substantially affect rhizosphere and bulk soil C and N contents (Becker et al., 2023). It is thought (Grönemeyer et al., 2014) that the decline in soil nutrients is most likely attributed to heat and drought conditions in recent years. Rhizobial inoculants, applied frequently as biofertilizers, play an important role in sustainable agriculture. Consequently, inoculation with an effective and suitable rhizobial strain for BGN might improve symbiotic nitrogen fixation and yield. It has been reported that inoculation also affects the microbial community by increasing the amount of the chosen rhizobial strain in the rhizosphere (Mohammed et al., 2021; Pulido-Suárez et al., 2021). Various researchers (Shin et al., 2016) have reported that inoculants could significantly improve yield in many leguminous crops. Moreover, the use of rhizobial inoculants for improvement in nitrogen fixation and productivity of grain legumes has been well-established in developed countries (Sajid et al., 2011).

For inoculant strain(s) to successfully establish themselves, they must be able to endure the soil environment and make use of the ecological niche that host plant roots supply (Ibny et al., 2019). Should non-competitive rhizobial strains be used for inoculation, they may be outcompeted by native rhizobial strains. This is supported by studies (Allito et al., 2021; Mendoza-Suárez et al., 2021) which show that some native rhizobia in the soil might be effective at root colonisation, and hence nodulation. In such circumstances, nodulation can be enhanced by inoculating seeds with more potent strains. This strengthens the hypothesis that strain competitiveness plays an essential role for successful inoculant establishment under field set ups. It might influence and deviate from competition experiment results obtained from growth chamber experiments with plants growing in sterile substrate like vermiculite.

Although several climate-smart, heat-tolerant native rhizobial strains have been isolated as pure culture from Namibia and in many cases defined as new species of *Bradyrhizobium* (Grönemeyer et al., 2014; Grönemeyer et al., 2015a; Grönemeyer et al., 2015b; Grönemeyer et al., 2016; Grönemeyer et al., 2017; Grönemeyer and Reinhold-Hurek, 2018), their development into inoculant to improve bean yield for subsistence farming in Namibia is just beginning; studies to determine the competitive strains to nodulate BGN roots and improve yields have not been carried out. Only for cowpea have climate-adapted indigenous strains been tested as bioinoculants to enhance the yield of Namibian varieties in the Kavango region (Luchen et al., 2018).

This study consequently seeks to explore the potential of different local *Bradyrhizobium* strains for root nodule symbiosis with BGN, in order to foster the development of inoculant strains for Namibia. Three differently colored Namibian BGN varieties (cream, red, brown) were used, provided by UNAM-ZERI, as seed coat color compounds appear to play a role in nodule development and nitrogen fixation (Redjeki et al., 2013; Puozaa et al., 2021) and the choice of microsymbiont partners (Ibny et al., 2019). We, therefore, assessed after seed inoculation with different *Bradyrhizobium* strains the nodulation, yield components, and competitiveness in laboratory and greenhouse conditions.

## 2 Material and methods

### 2.1 Study site and planting materials for pot experiments in-greenhouse

Topsoil for pot experiments was collected from the Mashare Research Station of the Ministry of Agriculture, Water, and Land Reform (GPS coordinates of 17° 54′ 08.13″ S and 20° 08′ 00.99″ E) located in Kavango East, from where some soil data already exists (Gröngröft et al., 2013). This soil had neither any previous records of growing pulses nor heavy fertilization. It was collected before the rainy season and was transferred to Windhoek in plastic bags at room temperature. Three Namibian BGN varieties (brown, cream, and red) were used. They were obtained from the Namibian Institute of Seeds (NIOS) and were locally selected and purified uniform lines (L. N. Horn, unpublished). The pot experiments were carried out in a greenhouse environment of the Zero-Emission Research Initiative (ZERI) at the University of Namibia’s main campus (GPS coordinates of 22° 36’ 23.99” S and 17° 03’ 16.20” E), with plant growth monitoring, watering, and photographic record. The plants were harvested and carefully uprooted after 50 days.

**Figure 1:**
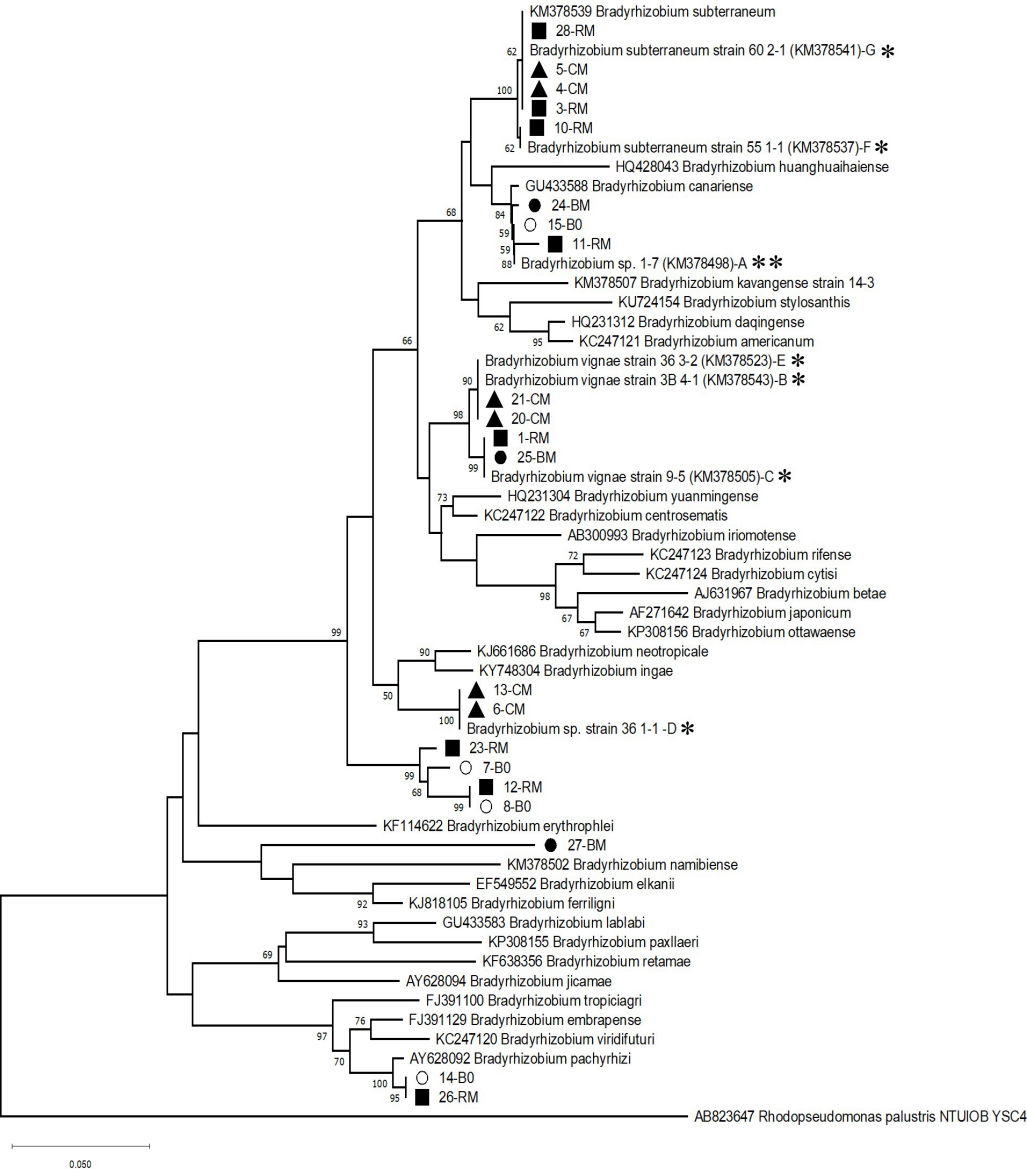
Phylogenetic tree representing the evolutionary relationships of the taxa of the strains of *Bradyrhizobium* spp. used in this study, based on 16S-23S intergenic spacer (ITS) sequences. Six of the individual strains of the mixed inoculant MK (B-G) originating from BGN nodules are denoted with *, strain A of the mix originating from peanut is denoted with **. Strains labeled with other symbols were isolated from root nodules induced on BGN in a greenhouse pot experiment in Kavango soil, without inoculation (B0, empty circle), or mixed inoculant of seven *Bradyrhizobium* strains (MK, filled black symbols). 16S-23S intergenic spacer (ITS) - fragments of *Bradyrhizobium* strains were amplified from DNA extracted from individual BGN root nodules collected from three independent pot experiments. The evolutionary history was inferred using the Neighbor-Joining method (Saitou and Nei, 1987). The percentage of bootstrap tests (1000 replicates) are shown next to the branches (Felsenstein, 1993), and values below 50% were not considered. The evolutionary distances were computed using the Maximum Composite Likelihood method (Tamura et al., 2004). This analysis involved 58 nucleotide sequences. The following type strains were selected to draw the tree are as follows: *Bradyrhizobium subterraneum* 58 2-1, *Bradyrhizobium huanghuaihaiense* CCBAU 23303, *Bradyrhizobium canariense* CCBAU 51257, *Bradyrhizobium stylosanthis* BR 446, *Bradyrhizobium daqingense* CCBAU 15774, *Bradyrhizobium americanum* CMVU44, *Bradyrhizobium kavangense* strain 14-3, *Bradyrhizobium yuanmingense* CCBAU 15773, *Bradyrhizobium centrosematis* A9, *Bradyrhizobium iriomotense* EK05., *Bradyrhizobium rifense* CTAW71, *Bradyrhizobium cytisi* CTAW11, *Bradyrhizobium betae* PL7HG1, *Bradyrhizobium japonicum* USDA 62, *Bradyrhizobium neotropicale* BR 10247, *Bradyrhizobium ottawaense* OO99, *Bradyrhizobium ingae* BR 10250, *Bradyrhizobium erythrophlei* CCBAU 53325, *Bradyrhizobium namibiense* 5-10, *Bradyrhizobium elkanii* USDA 76, *Bradyrhizobium ferriligni* CCBAU 51502, *Bradyrhizobium lablabi* CCBAU 23086*, Bradyrhizobium paxllaeri* LMTR 21, *Bradyrhizobium retamae* Ro19, *Bradyrhizobium jicamae* PAC68, *Bradyrhizobium tropiciagri* SEMIA 6148, *Bradyrhizobium embrapense* SEMIA 6208, *Bradyrhizobium viridifuturi* CMVU30, *Bradyrhizobium pachyrhizi* PAC48. There was a total of 461 positions in the final dataset. ITS sequence from *Rhodopseudomonas palustris* AB498825 served as an outgroup. Filled square, red variety (RM); filled circle, brown variety (BM); filled triangle, cream variety (CM); the number tagged to each nodule isolate represents the strain number. Empty circles represent nodule isolate(s) from the brown variety with no inoculant treatment (B0).

The bacteria used in this study were previously isolated from Namibia (Grönemeyer et al., 2014). The strains were *Bradyrhizobium* spp. 1-7, 36 1-1, *B. vignae* 36 3-2, 9-5 and 3B 4-1, and *B. subterraneum* 55 1-1 and 60 2-1 (**Figure 1**).

### 2.2 Bioinoculant preparation and application on beans for pot experiments

Peat is generally considered as “industry standard” carrier material in Biofertilizer production. The peat for biofertilizer preparation was Florbest Gartentorf; (Einheits-Erde, Sinntal, Germany), which is very acidic white peat with a pH between 3-4, containing 95% organic substance with 700-900% water holding capacity. The peat was oven-dried at 75° C for 24 h, homogenized, and pH neutralized by stepwise pH adjustment with calcium carbonate (CaCO_3_) to pH around 6.8-7. Finally, the neutralized peat was sterilized by autoclaving and dried overnight at 60°C in an oven, aseptically.

To load the carrier, pure cultures of individual inoculant strain(s) were grown up to an optical density (OD_600_) of 1 -1.2 in fresh liquid Modified Arabinose Gluconate (MAG) medium (van Berkum, 1990), in Erlenmeyer flasks with constant shaking at 30°C on an orbital shaker. After centrifugation under aseptic conditions at room temperature, cell pellets were suspended in fresh MAG medium to a final OD_600_ of 0.2. To generate one bag of biofertilizer, to 3.5 g of neutralized, sterile peat in a 20 ml sample bag from Whirl-Pak^®^ Nasco (Fort Atkinson, USA), 6.5 mL of culture was added, either as a single strain or as a mix of strains in equal proportions. Closed bags without air bubbles were kneaded from the outside so that it was well mixed without any leftover residual fluid. Finally, the bags were incubated at 30°C for 3-4 days for curing. They were transported to Namibia at room temperature and stored at 4°C until usage.

Before inoculation, BGN seeds were checked for integrity (avoiding boreholes of insects, etc). For inoculating seeds, 25 mL of water was mixed with the content of one bag of biofertilizer to make a slurry. Prior to planting it was mixed well with 150-200 g of BGN seeds. First, the respective pots with non-inoculated seeds wetted with water only were sown. Thereafter the inoculated seeds or seeds with other treatments were sown to prevent cross-infection with bacterial inoculants.

### 2.3 Experimental layout of pot experiments and data collection

Pot experiments were carried out with all three varieties of BGN (red, brown, and cream), **Figures S1A–C**. Soil from the Kavango region was used for pot experiments, which might still have indigenous strains, so that the competitiveness of the inoculant strain could be tested.

A photographic pictorial representation of the pots in the greenhouse at the University of Namibia main campus is depicted in **Figure S1D**. The experiment was performed in a randomized complete block design with a three-by-three factorial design under three replications. For each treatment with a single variety of Bambara groundnut (BGN) growth, three technical replicate pots were used in the greenhouse. Considering all three types of treatment including one without inoculant (0K), or with single inoculant strain 9-5 (9-5K), or mixed inoculant (MK) on three BGN varieties (red, brown, and cream), 27 independent pots represented one independent biological experiment. Data from three such biologically independent replications of the pot assays was collected (with 81 pots in total) and considered for further statistical analysis. The observation was done daily for 60 days and data on the parameters of interest was captured and analyzed (**Figures S1E–I**). All three varieties of BGN grew well in the pots within the greenhouse (**Figures S1E, D**). Parameters such as the number of nodules per plant, plant height (cm), and shoot weight were collected 60 days after planting for further analysis. Plant height was determined at 30 and 60 days after planting (DAP). Root nodules were examined at 60 DAPS. (**Figures S1F, G**), and sampled nodules were collected and dehydrated in silica gel-filled vials (**Figure S1H**) until analysis.

#### 2.3.1 Identification of rhizobia in nodules

Freshly harvested nodules from pot experiments were stored and transported in closed glass vials containing dehydrated silica gel stored at 4°C (Grönemeyer et al., 2013). Rehydration and surface sterilization of the nodules and subsequent isolation were carried out as described (Grönemeyer et al., 2013). Surface-sterilized nodules were crushed in sterile water; the extract was streaked on modified arabinose gluconate (MAG) agar plates (van Berkum, 1990) and incubated for a minimum of 7 ds at 28°C.

For identification of the nodule symbionts, the sequence analysis of the 16S-23S rRNA internal transcribed spacer (ITS) region was used. PCR amplification was carried out either from crude cell lysate of pure bacterial cultures (fast method successful for bacterial cultures) or from genomic DNA directly extracted from surface sterilized crushed nodules, which required a kit to remove potential inhibitory contaminations, using NucleoSpin^®^ Tissue kit (Macherey-Nagel, Düren, Germany, no. 740952.50) according to the manufacturer’s instructions. The ITS DNA fragment was amplified using the non-degenerate primer pairs: FGPS 130 (5’–CCGGGTTTCCCCATTCGG-3’: 18-mer; Tm 60.5°C) and FGPS 1490 (5’–TGCGGCTGGATCACCTCCTT-3’; 20-mer; Tm 61.4°C) as described by (Laguerre et al., 1996) with increased annealing temperature at 58°C.

Prior to sequencing, PCR products were purified using either the Nucleospin^®^ Gel- and PCR Clean-Up (Macherey-Nagel, Düren, Germany) or the Monarch^®^ Nucleic Acid Purification Kit (NEB, Ipswich, Massachusetts, United States). Custom Sequencing by the Sanger method was carried out by LGC Genomics (Berlin, Germany). The DNA sequences obtained in this study were submitted to GenBank, GenBank accession numbers are OR392289 to OR392309. 8-B0: OR392289, 23-RM: OR392290, 10-RM: OR392291, 7-B0: OR392292, 11-RM: OR392293, 13-CM: OR392294, 6-CM: OR392295, 14-B0: OR392296, 4-CM: OR392297, 15-B0: OR392298, 20-CM OR392299, 3-RM: OR392300, 1-RM: OR392301, 21-CM: OR392302, 12-RM: OR392303, 5-CM: OR392304, 24-BM: OR392305, 27-BM: OR392306, 28-RM: OR392307, 25-BM: OR392308, and 26-RM: OR392309. Phylogenetic analyses were conducted in MEGA 11 (Tamura et al., 2021). Alignments were generated by MUSCLE. DNA sequences of selected type species and reference strains were retrieved from GenBank.

### 2.4 Data analyses

The statistical software Genstat^®^ (14th edition, VSN International, UK) was used to determine significant differences between treatments from greenhouse pot experiments using the Analysis of Variance (ANOVA) function. The data were further subjected to mean comparisons and correlation analysis on all the traits.

### 2.5 Setting up growth chamber (Phytotron) experiments with BGN under aseptic conditions

For surface sterilization of seeds, after initial washing with water, BGN seeds were treated with 70% ethanol for a few seconds, washed with water several times, and incubated with 2.5% freshly prepared sodium hypochlorite for 8 mins. Finally, the seeds were washed with several changes of water and soaked overnight in sterile water. Individual seeds were then placed on 1% water agar and incubated in the dark at 28°C. Depending on the variety, the germination time ranged from 3-4 days to 12-14 days.

For inoculation with symbionts, individual strains of *Bradyrhizobium* were freshly grown in liquid MAG medium at 28°C and adjusted to a final OD_600_ of 0.2 (10^9^ cells of the respective isolate) in 1% (wt/vol) sucrose solution. Freshly germinated BGN seedlings were transferred to Magenta GA7 vessels filled with washed vermiculite, supplemented with 0.5x N-free Jensen’s medium (Jensen, 1942). For competition assays for nodule occupancy, individual strains (seven reference strains, see above) were mixed in equal proportion according to their optical density of 0.02 per strain shortly before the plant inoculation. Cells were always harvested from exponentially growing cultures (OD_600_ of 0.6–0.9) to ascertain viability. Each plant was inoculated with 1 mL of the mixture. The plants were maintained in a phytotron with day/night (11.5/12.5 h) temperatures of 28/25°C at 60% humidity. Plants were watered regularly with sterile distilled water. Three independent BGN inoculation experiments (biological replicates) were carried out with 3-4 replicate plants each, along with several negative control plants without inoculation and positive control plants treated with 5 mM potassium nitrate as an N source instead of inoculation, respectively. Plants were watered regularly and harvested 6-8 weeks after the first emergence of young shoots≥ 2 cm.

### 2.6 Assessment of plant growth and nitrogen fixation

In aseptic phytotron experiments, to measure the shoot dry weight of the plants, individual shoots were detached from the root and seed, oven-dried at 65°C for 3 days in paper bags, and then the individual weight was measured. From the harvested plants, root nodules were counted and recorded. Then individual roots with nodules were used for Acetylene Reduction Assay to assess the nitrogenase activity. Root systems including the nodules were cut off from shoots, incubated in 15 mL tubes sealed with rubber stoppers, and exposed to 10% [v/v] of acetylene for 4h at 30°C. The formation of ethylene was quantified by gas chromatography on an Agilent 7820A GC System, as described in (Bünger et al., 2021).

SPAD values (Soil-plant analysis development) are the measure of chlorophyll content of leaves. They were measured by a SPAD-502 (Minolta Camera Co., Osaka, Japan); the dates were recorded 6-8 weeks after inoculation (or uninoculated control plants) and just immediately before the harvest. SPAD values were measured from the three uppermost young, expanded leaves of each plant as described (Bünger et al., 2021).

In greenhouse experiments, plant height was determined by measuring the plant from the crown to the last leaf. Root nodules were examined at 60 days after planting (DAP). The roots were removed using a hand trowel without causing any nodule damage the number of young nodules was counted, and the total nodules per plant was recorded. Sampled nodules were then collected and dehydrated in silica gel-filled vials that were kept at 4°C until analyzed. Plant shoots were obtained by cutting out the root and the shoots were weighed immediately to determine the shoot’s fresh weight, and after a 72-hour oven drying period at 65°C, on an electronic balance to determine their dry weight.

### 2.7 Rapid identification of nodule symbionts from aseptic experiments by genomic fingerprints

Competitive nodule occupancy of each strain of the inoculant mixture was identified by a rapid test suitable for known bacterial isolates, applied to the pure cultures isolated from each nodule. Two repetitive PCR-mediated DNA fingerprinting tools were carried out, ERIC-PCR or BOX-PCR (De Bruijn, 1992; Schneider and de Bruijn, 1996). The primer used for BOX-PCR was BOXA1R 5’-CTACGGCAAGGCGACGCTGACG-3’ using the PCR conditions: initial denaturation at 95°C for 7 min followed by 35 cycles (94°C for 1 min, 53°C for 1 min, 65°C for 8 min) and one final step at 65°C for 16 min). ERIC-PCR was conducted using Forward primer: ERIC1R 5’-ATG TAAGCTCCTGGGGATTCAC 3’ and Reverse primer: ERIC2 5’-AAGTAAGTGACTGGGGTG AGCG-3’ and following the PCR condition: initial denaturation at 95°C for 7 min followed by 30 cycles (94°C for 1 min, 52°C for 1 min, 65°C for 8 min) and one final step at 65°C for 16 min (Versalovic et al., 1994; Schneider and de Bruijn, 1996). Electrophoresis was carried out in 1.5% agarose gel in 1X TAE buffer for 70 min at a constant 90 V.

To validate the equal proportion of the seven strains in the mixed inoculum, the biofertilizer was plated, and colonies (n = 21) were picked randomly and identified by ERIC-PCR. In that way, a correction factor could be allocated to each individual strain of the mix when they were not found at an equal ratio in the starter mix. After competition and nodule occupancy, strains were again cultivated from surface-sterilized nodules (see above), and randomly picked nodule isolates (n=21 nodules per treatment) were identified by ERIC-PCR. In that way, a correction factor could be allocated for each individual strain of the mix which was used to calculate the comparative variable factor or Change factor (V) for each strain. V is the change in the proportions of the strains examined in the inoculum sample versus the nodule sample for all biological replicate samples. If the ratio of occurrence of a strain in the inoculum sample to that in the nodule sample was < 1, the change factor was initially assigned as Negative. For ease of presentation, negative change factors were assigned as zero. Change factors from each of the three independent experiments were averaged to obtain the overall mean enrichment or depletion for each strain, presented as a percentage of the mix.

## 3 Results

### 3.1 Selecting for putative climate-adapted *Bradyrhizobium* strains to be used as potential bioinoculants for Bambara groundnut cultivation in the Kavango region of Namibia

Strains to be tested as potential bioinoculants on the three BGN varieties were initially selected from a collection of strains isolated from Namibia (Grönemeyer et al., 2014). The following selection criteria were addressed: isolated from nodules of BGN as a host plant (except one, from peanut), hosts growing in Namibian soil of Mashare Agricultural developmental institutes or Kalahari sand, or old flood plains with either dryland agriculture practices or fallow land in Kavango East. With respect to ITS sequences, the isolates were phylogenetically diverse, and distributed over both major clades. Moreover, each of the isolates was chosen to show unique DNA fingerprint patterns well distinguishable from each other: in that way, they could be easily differentiated from each other in a mixed inoculant. Additionally, several of them exhibited heat tolerance until 38°C, drought tolerance with growth in 20% PEG, effectively nodulated BGN, and even cowpea. Several of them were further confirmed for their ability to nodulate local Namibian BGN varieties in aseptic growth chamber experiments (authentication). In this way, seven individual strains of *Bradyrhizobium* spp. were chosen as test strains that might be used as bioinoculants for BGN farming. The following **Table 1** represents the strains used individually and in the mixture of seven strains. *Bradyrhizobium* sp. 1-7 (designated A) was originally isolated from peanut and already performed very successfully as a cowpea inoculant in field studies in Mashare (Luchen et al., 2018). It was included as a cross-inoculant for BGN. A cross-inoculant originating from peanut has been already reported to enhance the yield of field-grown BGN in Chad and Cameroon (Gomoung et al., 2017).

**Table 1:** Selected *Bradyrhizobium* strains used in this study and their characteristics^a^ COW BAM HYA PEA PHA.

Species	Strain	Tag	Plant origin	Country origin	Soil type origin	Land use	Max. Temp. (°C) Solid^c^	Max. Temp. (°C) Liquid^c^	Max. PEG (%)	Nodulation of COW BAM HYA PEA HA				
*Bradyrhizobium* sp.	1-7	A	PEA^b^	NAM	MADI	DA	38	35	20	AN	AN	BN	BN	BN
*Bradyrhizobium vignae*	3B 4-1	B	BAM	NAM	OFP	F	38	40	ND	AN	AN	ND	ND	ND
*Bradyrhizobium vignae*	9-5	C	BAM	NAM	KS	DA	38	40	20	AN	AN	ND	ND	BN
*Bradyrhizobium vignae*	36 1-1	D	BAM	NAM	KS	DA	35	35	20	AN	AN	AN	AN	BN
*Bradyrhizobium vignae*	36 3-2	E	BAM	NAM	KS	DA	38	40	ND	AN	AN	ND	ND	BN
*Bradyrhizobium subterraneum*	55 1-1	F	BAM	NAM	OFP	DA	38	35	20	AN	AN	BN	AN	BN
*Bradyrhizobium subterraneum*	60 2-1	G	BAM	NAM	OFP	DA	38	38	ND	AN	AN	AN	AN	BN

a Data from (Grönemeyer et al., 2014) and (Grönemeyer et al., 2015a).

b ND, not determined; COW, cowpea; BAM, Bambara groundnut; HYA, hyacinth bean; PEA, peanut; PHA, common bean; NAM, Namibia; MADI, Mashare Agricultural Development Institute; KS, Kalahari sands; OFP, old floodplain soil; DA, dryland agriculture; F, fallow after subsistence farming; AN, effective nodulation often measured with ARA; BN, ineffective nodulation.

c Maximum temperature for growth on solid or liquid medium, respectively.

### 3.2 Effect of bioinoculant treatment on agro-morphological traits in red, brown, and cream variety of BGN in pot experiments

For pot experiments, the three varieties of BGN (red, brown, and cream) were individually inoculated either with a mix of seven reference strains in equal proportion (MK) to check for competitive root nodule occupancy, or they were not inoculated (negative control, 0K). Strain 9-5 (designated as C) was additionally included as a single inoculant because it showed promising features in a preliminary aseptic experiment in the phytotron (**Figure S2**): Using a local undefined landrace of BGN and only four *Bradyrhizobium* strains (A, 1-7; C, 9-5; D, 36 1-1; G, 60 2-1), single strain inoculation resulted in enhanced chlorophyll fluorescence (SPAD-value **Figure S2A**) and shoot dry weight (**Figure S2B**) particularly by strain 9-5; additionally, strain 9-5 was highly competitive in the mixture of four strains, occupying 87% of the root nodules (**Figure S2C**).

The analysis of variance for days to emergence, plant height, days to flowering, number of nodules per plant, shoot fresh weight, and shoot dry weight of Bambara groundnut varieties is shown in **Table S1**. The results indicated significant interactions (P ≤ 0.05) in most of the agronomic traits studied (**Table S1**). The mean square values and significance levels (P ≤ 0.05) are indicated in the analysis of variance (ANOVA) tables to discern significant differences. Corresponding means and ranges, standard errors, least significant differences, and coefficient of variations for various traits are presented in **Tables 2**, **S2**.

**Table 2:** The mean values for agro-morphological traits of three Bambara groundnut (BGN) varieties treated with *Bradyrhizobium* strains as inoculants evaluated in pots under greenhouse conditions.

Variety	Inoculant	DTE	PHT (cm)		DTF	Nodule number/plant	SFW (g)	SDW (g)
			30DAP	60 DAP				
Brown	0K	11.30^bc^	13.90^b^	14.60^bc^	60.70^abc^	3.70^bc^	6.00^e^	0.90^g^
	9-5K	9.00^d^	14.50^ab^	14.30^bc^	62.00^ab^	7.70^ab^	6.50^d^	1.00^f^
	MK	10.70^bc^	14.50^ab^	12.20^c^	59.30^bc^	7.70^ab^	9.20^b^	1.20^c^
Mean		10.3	14.3	13.7	60.7	6.3	7.2	1.0
Cream	0K	10.00^cd^	15.30^ab^	15.60^abc^	62.70^a^	0.00^c^	7.70^c^	1.00^e^
	9-5K	8.70^d^	14.10^ab^	14.30^bc^	60.00^abc^	5.70^abc^	6.00^e^	0.90^h^
	MK	10.00^cd^	17.10^ab^	18.00^ab^	58.00^cd^	8.70^ab^	10.00^a^	1.70^a^
Mean		9.6	15.5	16.0	60.2	4.8	7.9	1.2
Red	0K	14.00^a^	16.80^ab^	17.70^ab^	55.70^d^	0.00^c^	7.30^c^	1.10^d^
	9-5K	11.70^b^	15.80^ab^	17.80^ab^	56.30^d^	7.30^abc^	7.40^c^	1.10^d^
	MK	12.00^b^	17.80^a^	19.20^a^	56.00^d^	13.00^a^	10.00^a^	1.50^b^
Mean		12.6	16.8	18.2	56.0	6.8	8.3	1.2
Grand mean		10.8	15.5	16.0	59.0	6.0	7.8	1.2
SED		0.75	1.69	1.87	1.33	3.51	0.19	0.01
LSD (5%)		1.59	3.59	3.96	2.82	7.44	0.40	0.03
CV (%)		8.5	13.4	14.3	2.8	72.0	2.9	1.5

0K, no inoculant; 9-5K, inoculated with strain 9-5; MK, mixed inoculant of seven strains from **Table 1**;

DTE, Days to emergence; PHT, plant height; DAP, Days after planting; DTF, Days to flowering;

Nodule_plant, Number of nodules per plant; SFW, Shoot fresh weight; SDW, Shoot dry weight;

SED, Standard error of difference; LSD, least significant difference; CV, coefficient of variation; Values followed by the same letters are not statistically different LSD test at P < 0.05.

The nodulation differed in the varieties (**Table 2**). In Kavango soil without inoculant treatment (0K), root nodules were detected only for the brown variety and not for red or cream. Apparently, indigenous strains occurred in the Kavango soil which are specific for the brown variety.

Inoculants MK or 9-5K induced root nodulation in all other varieties. For the brown variety, nodulation was equally increased by both inoculants. In contrast, the inoculant treatment with strain 9-5 and MK differed in their ability to induce root nodules in red and cream BGN variety; the inoculant mixture induced more nodules than the single inoculant strain 9-5, with the highest number in the red variety. Statistical analysis of nodule numbers showed there were significant differences across the means of the mixed inoculant on red or cream varieties, respectively compared to uninoculated controls at (P < 0.05). On the contrary, there were no statistically significant differences recorded among the means of the variety and among the interaction of treatment and variety with nodule number per plant (**Table S1**).

With respect to plant growth, several trends of inoculant effect were found at 60 DAP (days after planting). The red variety had the tallest shoots, with a mean height almost 40% larger than those of the brown variety considering all the treatments. With respect to treatments, inoculation did not influence plant height significantly in any variety, although the trends matched with the shoot biomass (dry weight). Mixed inoculum increased the plant height slightly in the red variety. Also for the brown variety, the average shoot height was not increased by inoculation, although more nodules could be detected with both types of inoculant treatments. Thus, increased nodulation was not correlated with plant height. Shoot dry mass data were more explicit. Shoot dry mass of the brown variety increased only slightly with 9-5K treatment compared to uninoculated controls, whereas it increased significantly by 33% with MK treatment (**Table 2**). In contrast, for the cream variety, the shoot dry mass decreased slightly by strain 9-5, whereas a strong increase of 70% was obtained with mixed inoculant (**Table 2**). Also in the red variety, the mixed inoculant yielded a strong increase by 50%, while the single strain had no effect (**Table 2**) Thus, not strain 9-5 but the other strain(s) within the mix or other indigenous strains from the soil might be more efficient and responsible for shoot biomass enhancement.

No statistically significant difference in plant height could be observed after 30 DAP among the varieties and in the interaction between bioinoculant treatment and variety (P < 0.05). Similarly, no significant difference could be observed in plant height after 60 DAP among bioinoculant treatment or bioinoculant treatment x variety interaction. However, plant height varied significantly depending on variety at 60 DAP (P < 0.01). The statistical analysis of shoot fresh weight and shoot dry weight revealed that there were statistically significant differences at (P < 0.05) among varieties, treatments, and the treatment-variety interaction (**Table S1**). Comparing all varieties and treatments, the cream variety with MK treatment had the highest shoot dry weight (1.71 g plant^-1^) (**Table 2**).

Independent from treatment, the time until the emergence of plants was relatively long for the red variety (12.6 days), compared to the brown and cream varieties (10.3 and 9.6 days, respectively); it appears to have a longer germination time. For the brown variety, the 9-5K treatment reduced the germination time significantly. On the other hand, for the red variety, both the inoculation treatments (single or mixed) reduced the germination time significantly. The cream variety showed no significant influence on germination. Although late germinating, the red varieties were relatively early flowering (56 days) compared to other varieties without any significant differences between the varieties and treatment.

**Table S2** presents pair-wise correlation coefficients among agro-morphological traits with their levels of significance. In both cases, plant height at 30 days after planting (DAP) had a positive and significant association with shoot fresh weight (0.726), and shoot dry weight (0.796) at 0.05 level, respectively. Moreover, days to emergence (DTE) were negatively correlated (-.737) with days to flowering (DTF) at a 0.05 level of significance.

### 3.3 Phylogenetic characterization of rhizobia from nodules of greenhouse pot experiments in Kavango soil

In order to assess whether and which of the inoculated rhizobia established inside root nodules and could thus compete against native rhizobia in the Kavango soil, sequence analyses were carried out. From individual pure cultures of the nodule isolates, the ITS region was amplified by PCR. Sequencing and blast analysis of ITS-PCR products revealed that all of them belonged to the genus *Bradyrhizobium* as expected. The ITS sequences of all the nodule isolates obtained from three BGN varieties either with MK or 0K treatments were aligned with individual seven reference strains of MK as well as with additional type strains from different *Bradyrhizobium* species. The phylogenetic position of the bacterial isolates was evaluated by constructing a phylogenetic tree using the Neighbor-Joining method (**Figure 1**). The distribution of six of the seven diverse reference strains included in the mix followed the same pattern in the phylogram that had been previously generated by (Grönemeyer et al., 2014) forming distinct clusters. Strain 36 1-1(D) which was recently included in the phylogram formed a distinct cluster, too, closely related to *B. vignae*. Nodule isolates from red, brown, or cream varieties were designated as RM, BM, or CM (**Figure 1**). Several of them present in the mix were clustering together with the reference strains. Therefore, the reference strains of the mix could successfully establish themselves in the nodules of the three varieties. The highest number of nodule isolates was associated with the cluster from *B. subterranean* 60 2-1 (G), showing its high nodule-competitive ability compared to others for the red and cream variety but surprisingly not for the brown variety. Additionally, several CM isolates clustered together with either D (36 1-1) or B and E (36 3-2 or 3B 4-1) show strong affinity of these reference strains towards the cream variety. For the red variety, several of the nodule isolates belonged to a unique, deep-branching cluster along with nodule isolates obtained from the brown variety (7-B0, 8-B0). They probably comprise novel indigenous strains which nodulate the brown variety without inoculant treatment (B0), and additionally outcompete the reference strains of the mix to occupy the nodules of the red variety. This novel cluster does not represent any described type species of *Bradyrhizobium*. Other collected nodule isolates from the brown variety (BM) with MK treatment grouped together either with reference strain A or C, indicating poor nodule establishment of the other reference strains of the mix with the brown variety.

### 3.4 Plant growth promotion of BGN varieties by single inoculant strains under aseptic conditions in phytotrons

**Figure 2:**
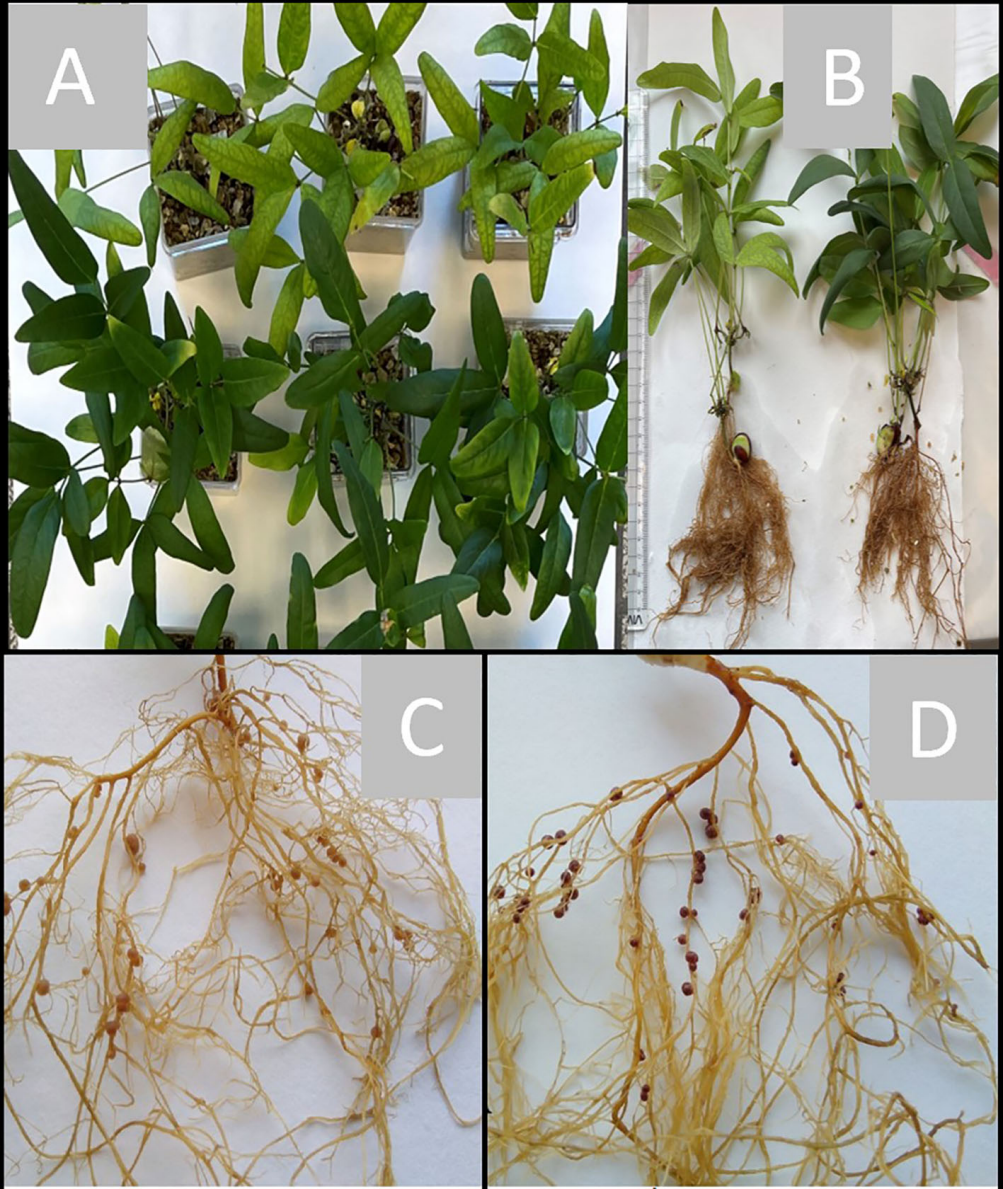
Representative diagram depicting growth and root nodulation of the red variety of BGN growing under aseptic conditions in vermiculite in phytotron, in response to inoculant treatment at the time of harvest. **(A)** BGN-red variety after six weeks of growth and just before harvest; first three plants on top: non-inoculated; three at the bottom, single strain inoculation with strain 9-5. **(B)** Harvested inoculated plant with nodules (right) compared to non-inoculated one without root nodulation (left). **(C, D)** Root nodulation of red variety of BGN after 6 weeks of inoculation with either strain 36 3-2 (= E) **(C)**, or strain 55 1-1 (= F) **(D)**. Red, pink, and purple colored representative root nodules after inoculated with a reference strain 36 3-2 in **(C)**, compared to 90% root nodulation with unusual dark purple colored, pigmented coloration when inoculated with 55 1-1 in **(D)**.

**Figure 3:**
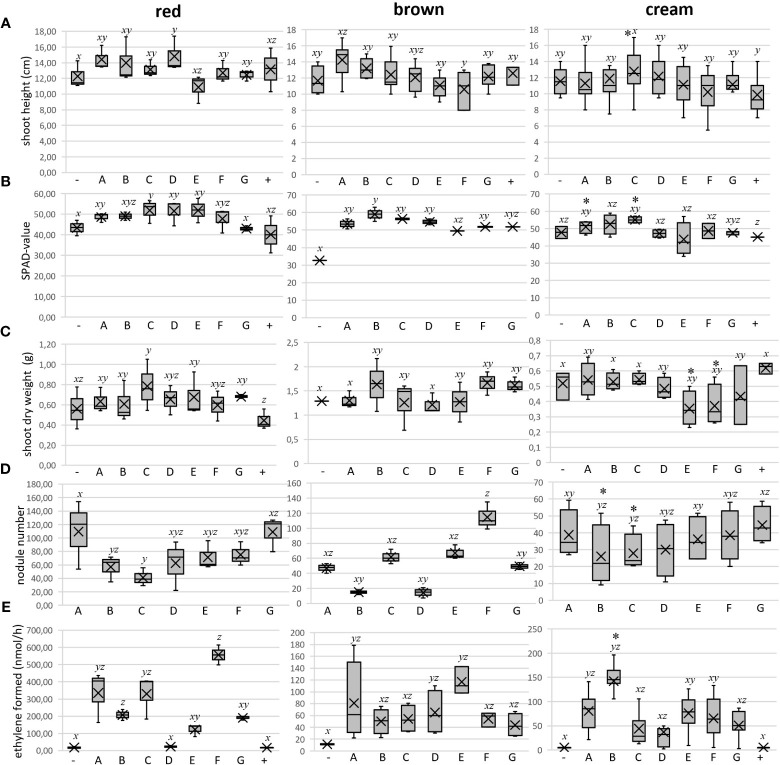
Measurements of several plant growth parameters as well as root nodulation and nodule nitrogenase activity of the three BGN varieties-red (left panel), brown (middle panel), and cream (right panel) in response to inoculant treatment and incubated for 6-7 weeks in Phytotron, along with controls. The results are represented as box plots: -, negative control with no inoculant, no nitrogen; A, 1-7; B, 3B 4-1; C, 9-5; D, 36 1-1; E, 36 3-2; F, 55 1-1; G, 60 2-1; +, positive control with 5 mM potassium nitrate as N source). **(A)** mean shoot length of the plants in cm; **(B)** mean SPAD value of the leaves of the plants; **(C)** mean shoot dry weight of the plants in gram; **(D)** root nodulation represented as mean number of nodules; **(E)** mean nitrogen fixation activity within the root nodules measured by the amount of ethylene formed in nmol per hour by Acetylene Reduction Assay (ARA). [n = three independent biological replicates consisting of 1-3 plants each. Representation of the median as a black bar, the 1st and 3rd quartiles as a box, and the minimum and maximum values as whiskers. The overall mean is shown as a cross]. Respective data for each parameter were initially checked for normality (Gaussian distribution) and homoscedasticity (=equality of variance). In order to compare the multiple data sets and to know if a relation exists between them, the one-way ANOVA (Analysis of variance) test and subsequent posthoc analysis by Tukey’s test were carried out using R statistical computing platform from R Foundation for Statistical Computing, Vienna, Austria. The Agricolae package was installed before running Tukey’s *post-hoc* test. In general, data of treatments with different letters indicate statistical significance (p<0.05). While performing ANOVA and considering analysis from single plant data and not as mean from any treatment specifically with cream variety (right panel), weak significant differences when observed are denoted additionally as * above each respective boxplot.

To test the effects of individual inoculant strains on BGN growth, short-term experiments were carried out in an artificial, sterile substrate (vermiculite) under controlled conditions of a phytotron. Here, plant parameters were recorded within 6 to 8 weeks after inoculation, and in addition to a negative control without inoculant, a positive control fertilized with potassium nitrate but without inoculant was included. In most of the cases, the inoculated plants were visually healthier and greener at the time of harvest compared to the uninoculated negative controls across independent experiments, which is a preliminary indication of root nodule symbiosis and nitrogen fixation (**Figure 2A**). Only the inoculated plants had root nodules compared to those of non-inoculated ones or those treated with inorganic nitrate as N-source (**Figure 2B**). However, variation was observed with respect to the measured plant growth parameters in response to single bacteria treatment. For simplicity, strain designations are here depicted by single-letter alphabetic characters. Overall, inoculation led to a slight improvement in plant growth parameters compared to the controls in the red variety (**Figures 3A–E**, left panel). In comparison to the uninoculated control, the mean plant height was not significantly increased except by inoculant D (36 1-1) (**Figure 3A**, left panel). SPAD values as a measure for chlorophyll content and thus nitrogen status were mostly enhanced by inoculation, albeit significantly only by inoculant C (**Figure 3B**, left panel). This correlated with shoot weight increase, which was also significant for strain C (9-5) only (**Figure 3C**, left panel).

The root system was successfully nodulated upon inoculation with all bacteria, and nodules were further surveyed for number, color, morphology, and size. Different coloration is determined by the leghemoglobin content of the nodule, here only visually. The nodules analyzed were mostly round determinate type with a reddish color (**Figures 2C**, **S1G**). Strains A (1-7) induced the highest number of nodules, significantly different from the lowest number induced by strain C (9-5) (**Figure 3D**, left panel), although plant shoot response was strongest. Surprisingly 90% of the total nodules (75) inoculated with strain F (55 1-1) developed a very specific coloration. Instead of a reddish hue, they were dark purple in color, whereas the other residual nodules total did not show this coloration and were mostly small (**Figure 2D**). The nitrogenase activity within the root nodules was assessed by the Acetylene Reduction Assay (ARA), indicative of the activity of the enzyme nitrogenase using an artificial substrate of acetylene converting it into ethylene and subsequent quantification by gas chromatography (**Figure 3E**, left panel). Inoculation with F (55 1-1) resulted in the highest nodule nitrogenase activity (ethylene formed by the entire nodulated root systems), significantly different from inoculations with B (3B 4-1), D (36 1-1), E (36 3-2), and G (60 2-1). The lowest acetylene reduction was found with D (36 1-1), at 23.04 ± 6.16 nmol/h hardly differing from the values of the controls.

When comparing nitrogenase activities with the observed nodule colors, it was surprising that the dark purple nodules formed by strain F (55 1-1) had by far the highest acetylene reduction rates (**Figure 3E**, left panel). On the contrary, inoculation with strain C (9-5) induced a relatively high nodule nitrogenase activity with comparatively a smaller number of nodules, including no red nodules. On the other hand, E (36 3-2) and G (60 2-1) with a relatively large number of red nodules, had a rather low nitrogen fixation activity. The lowest nitrogenase activity was activity was recorded for strain D (36 1-1) with 50% white nodules.

The brown variety reacted partially differently to the inoculants. Overall, inoculation did not lead to a drastic improvement in plant growth parameters compared to the controls. With respect to plant height, A (1-7) was the best performer, however with significance only better than strain F (55 1-1) (**Figure 3A**-middle panel). In general, inoculant treatment enhanced the leaf SPAD values of the brown variety compared to the non-inoculated negative control, with strain B performing the best and with the most significance (p<0.05) compared to the negative control (**Figure 3B** -middle panel). However, the mean shoot dry weight of this variety was less affected by individual inoculant treatments, with the best (but only slight) non-significant improvement by strain B (**Figure 3C**-middle panel).

As expected, individual inoculation treatment evoked successful root nodulation of different numbers, colors, morphologies, and sizes on the brown variety. Like in the red variety, 90% of the nodules inoculated with F (55 1-1) developed were dark purple in color. The highest number of nodules was elicited by F, with significantly lower nodule numbers evoked by strains B (3B 4-1), D (36 1-1), and G (60 2-1) (**Figure 3D**-middle panel). In general, the mean nodule nitrogenase activity observed for the brown variety was much lower (roughly 5 times) compared to the red variety. Still, inoculant E (36 3-2) showed a trend of highest nitrogenase activity (with a lot of red nodules), albeit without statistical significance towards other strains like G (60 2-1) with the lowest (**Figure 3E**-middle panel).

Unlike the red and brown varieties, the cream variety appeared healthier and greener compared to uninoculated control only with certain single inoculants, like strain A (1-7). Both shoot height and shoot dry weight gave no indication of significant differences between inoculation and negative control (**Figures 3A, C**, right panel); only the shoot height of plants treated with D (36 1-1) and shoot dry weights of plants treated with strain C (9-5) and A (1-7) were slightly, but significantly higher than the fertilized positive control. Similarly, the SPAD values did not differ from each other significantly (**Figure 3B**, right panel). On the other hand, in addition to higher nodule weight (data not shown), plants inoculated with strain B (3B 4-1) exhibited an increased nitrogen fixation rate, albeit only very weakly significant. Strikingly, treatments with strains B (3B 4-1) and C (9-5) tended to have lower numbers of nodules.

Like in red and brown varieties, the root nodules of the cream variety inoculated with F (55 1-1) were exclusively dark purple in color. Therefore inoculating with strain F is exclusively responsible for eliciting purple coloration of nodules, independent of varieties. However, the number of nodules did not differ significantly between the rhizobial strains used (**Figure 3D**, right panel). Analysis of nitrogen fixation rates also revealed no differences (p = 0.666), however with trends of strains A (1-7) and B (3B 4-1) showing the highest nitrogenase activities (**Figure 3E**, right panel).

The ANOVA tests performed showed that plants treated with strain B (3B 4-1) had a high weight per nodule compared to other groups (data not shown). Analyses of the single plant data set continued to show a high nitrogen fixation rate and low nodule number for this strain (**Figures 3D, E**). In the single plant data set, A (1-7) -treated plants had higher SPAD values than the negative control. It also assigned several positive traits for C (9-5). After inoculation, the plants produced a low number of nodules but a high weight per nodule. In addition, they showed both significantly more leaves (data not shown) and higher SPAD values than the plants of the negative controls.

### 3.5 Nodulation competitiveness of potential inoculants under aseptic conditions in phytotrons

For the application of inoculants, they should not only fix nitrogen efficiently in symbiosis but also compete against other, indigenous rhizobia for nodule occupancy. Therefore, we addressed the question of which of our potential inoculant strains were most competitive in comparison to each other, in our aseptic test system in vermiculite. We therefore mixed seven strains in equal proportions, inoculated BGN varieties, and identified the strain occupying the induced nodules.

Repetitive (Rep)-PCR-mediated fingerprinting was used as a tool to identify the rhizobial competitors from the mix. Two types of Rep-PCR tools, BOX-PCR and ERIC-PCR, were initially tested on the reference strains to compare the sensitivity and reproducibility of two DNA fingerprinting techniques. The PCR-amplified fragments were size fractionated through a gel matrix to yield complex fingerprint patterns. Highly similar banding patterns (fingerprint) were obtained from strains E and B using BOX-PCR, thus they were difficult to distinguish from each other (**Figure S3A**). In contrast, the bands were more distinct and clearly distinguishable from each other when using ERIC-PCR (**Figure S3B**). Therefore, ERIC-PCR and not BOX-PCR was chosen as a tool to identify nodule isolates of BGN.

**Figure 4:**
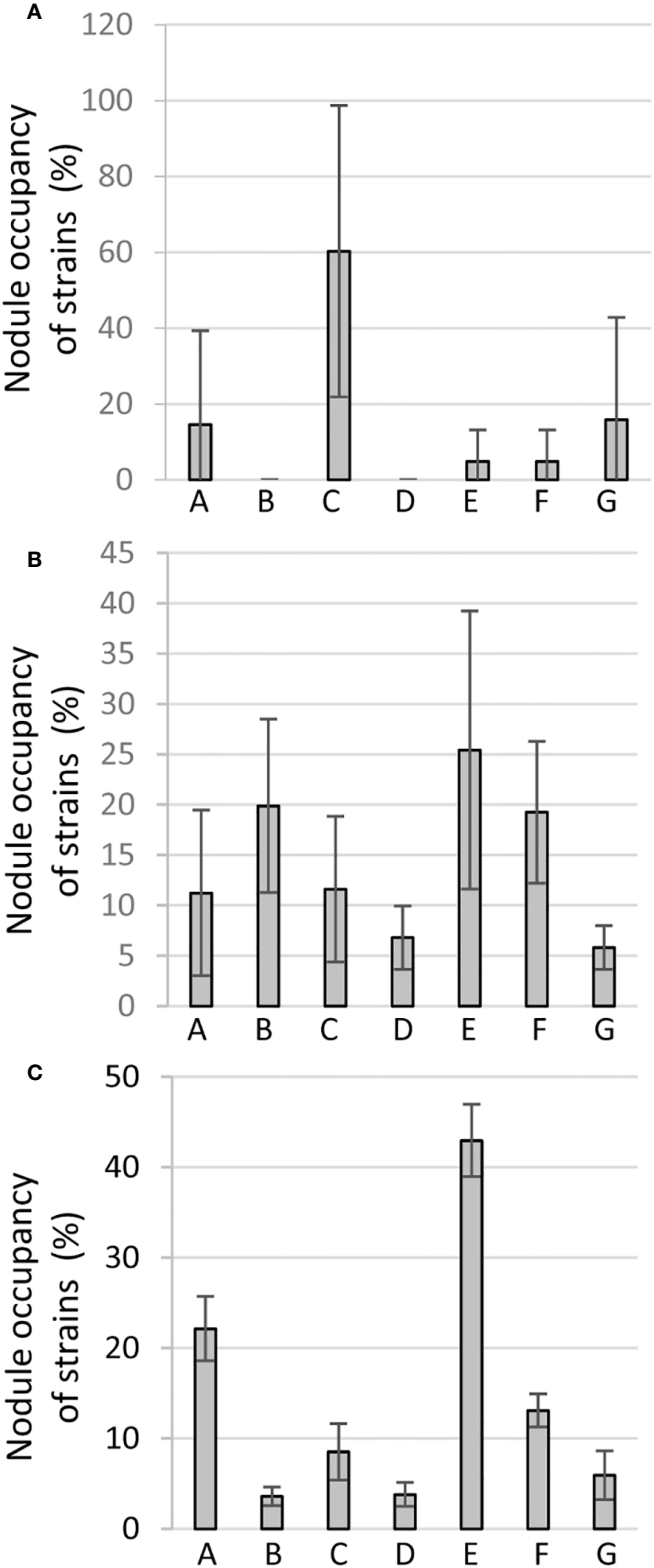
Competitiveness for nodule occupancy in red **(A)** brown **(B)** or cream **(C)** variety inoculated with the mix inoculant of equal proportion of each of the seven reference strains in Phytotron: A 1-7; B 3B 4-1; C 9-5; D 36 1-1; E 36 3-2; F 55 1-1; G 60 2-1. For a particular variety, the mean change factor converted to a percentage of each of the strains of the mix from three independent biological replicates (each with 2-3 technical replicate plants) is represented as a bar diagram with standard errors. The details of the calculation are described in Materials and Methods.

For the red variety, strain C (9-5) was most competitive with 60% of the nodules being occupied, followed by strains G(60 2-1) and A (1-7) occupying 16% and 15% of the total nodules, respectively (**Figure 4A**). Whereas others were weakly represented in nodules, B (3B 4-1) and D (36 1-1) were not detectable in the nodules of the red variety across all three biological experiments, and therefore considered probably least competitive for red variety (**Figure 4A**). The latter was also not well-performing as a bioinoculant for this variety in single inoculant experiments above.

For the brown variety, inoculant E (36 3-2) was found to be most competitive (25%) for nodule occupancy, albeit not as clearly, since strains B (3B 4-1) or F (55 1-1) also occupied 20% or 19% of the nodules, respectively (**Figure 4B**). Other reference strains were weakly represented with strain G (60 2-1) as the least competitive.

For the cream variety, strikingly, strain E (36 3-2) once more turned out to be the most competitive (43%) nodule occupant like in the brown BGN, followed by strain A (22%), whereas strains B (3B 4-1) and D (36 1-1) were weak competitors (**Figure 4C**). Interestingly *Bradyrhizobium* B and D were also weakest competitors and even unrepresented in the red variety, whereas strain B turned out to be the next best competitor for the brown variety.

## 4 Discussion

Here we studied competitiveness and plant growth improvement of Namibian *Bradyrhizobium* strains towards the development of inoculant for Bambara groundnut in Namibia for the first time. As there were no native soybean-nodulating bacteria in UK soils, soybean required inoculation with commercial elite *Bradyrhizobium* strains to exploit its BNF potential there (Maluk et al., 2023). In most other agronomic settings, inoculant formulation consisting of effective indigenous rhizobia being adapted to the local conditions may exhibit an improved performance. Studies have shown that there was a continuous increase in yield and quality of seeds when BGN was inoculated with local strains of *Bradyrhizobia* (Laurette et al., 2015). In a previous study on the characterization of rhizobial communities indigenous to the Namibian Okavango region, isolates were obtained from nodules of local varieties of pulses like cowpea, BGN, peanut, hyacinth bean, and common bean. Isolates from the semiarid sampling sites in Namibia were found to be mostly slow-growing species of *Bradyrhizobium* (Grönemeyer et al., 2014), and were very diverse, sharing an exceptionally high temperature tolerance (Grönemeyer and Reinhold-Hurek, 2018). They were a potential resource for inoculant development. Up to now, little information is available on the biodiversity of rhizobia nodulating BGN in African soils. Amongst at least 10 species of *Bradyrhizobium* spp. nodulating BGN in the laboratory, there are several that were Namibian isolates recently described as novel species, such as *B. subterraneum* (Grönemeyer et al., 2015a), *B. kavangense* (Grönemeyer et al., 2015b), *B. namibiense* (Grönemeyer et al., 2017), or *B. vignae* (Grönemeyer et al., 2016). Thus, this host plant appears to be quite promiscuous towards *Bradyrhizobium* spp., suggesting that inoculants that are well-adapted to varieties and environmental conditions may have to be developed for maximum benefit of yield. Similar observations were made for the orphan Kersting’s Groundnut in African soil where different bradyrhizobial populations are dominant symbionts of this legume across diverse agroecologies in Africa (Mohammed et al., 2021). Since Bambara is a native crop to Africa, an abundance of native rhizobia that are well adapted and capable of forming symbiotic relationships with the crop to efficiently fix N may be available in local soils. It is suggested that temperature resistant *Bradyrhizobium* strains should be isolated and selected on local soils (Grönemeyer and Reinhold-Hurek, 2018).

The *Bradyrhizobium* strains that we tested induced nodulation in all three Namibian varieties. This was congruent with the findings of Chen (Chen et al., 2018) who reported that inoculation enhances the number of nodules produced by ensuring that the presence of the desirable rhizobial strains is close to the root of the plant. However, we observed nodules also observed in the non-inoculated brown variety, which provided evidence of the existence of resident rhizobia in the Namibian Kavango soil used for the pot experiments. The use of native *Bradyrhizobium* strains for inoculation and the likely low population densities of native soil-borne bradyrhizobia could have contributed to the positive results obtained in the study. Studies carried out in the savanna region of the Benin Republic revealed that the indigenous rhizobia populations in the soil were inversely connected to the Bambara groundnut reaction to inoculation and that only response was shown when the population was < 5 rhizobia cells/g soil (Yakubu et al., 2010).

Moreover, the differences in nodule number per plant among the three varieties of Bambara groundnut indicated that the tested BGN varieties respond differently to the different *Bradyrhizobium* strains. This result confirms earlier reports that seed coat compounds have been noted to play a significant role in nodule formation (Redjeki et al., 2013; Ibny et al., 2019; Puozaa et al., 2021).

Our pot experiments revealed that the shoot dry weights were increased in the order of cream, red, and brown variety treated with mixed *Bradyrhizobium* inoculant. These findings are similar to previous studies (Gomoung et al., 2017) reporting that inoculation of BGN with rhizobial strains induced a considerable increase in plant length, fresh weight, and dry weight. Additionally, it was noted (Gedamu et al., 2021) that rhizobial inoculants enhance plant dry weight. It was observed that inoculating plants with *Bradyrhizobium* strain CB756 and (NC-92) peat-based commercial inoculants led to considerably increased dry matter yields in South Africa and northern Punjab, Pakistan, compared to un-inoculated controls (Latif et al., 2014; Chidebe et al., 2018). On the other hand, no increase in shoot biomass was observed in cream and brown variety treated with a single strain inoculant 9-5 in pot experiments. Probably the introduced inoculant strain 9-5K did not induce sufficient nodule N_2_ fixation at the time of sampling or might have failed to colonize the roots. Interestingly, treatment with strain 9-5K reduced the germination time (DTE) of all the Bambara groundnut varieties in pot experiments compared to the non- inoculated ones: For the brown variety, 9-5K treatment alone and for the red variety, 9-5K as well as MK treatment (containing also strain 9-5) reduced the time of seed germination (DTE) significantly. Therefore, it appears that strain 9-5 might act additionally as a biostimulant and was probably responsible for faster germination of Bambara groundnut seeds, a function yet not been explored.

The identification of nodule-inhabiting rhizobia in pot experiments in Kavango soil suggests differential competition and nodule occupancy of tested strains depending on the BGN variety. For example, *Bradyrhizobium* clusters of inoculant strains 3B 4-1, 36 1-1, and 36 3-2 were not represented in nodules of the red variety, whereas inoculants 1-7, 9-5, and 55 1-1 were not detected in nodules of the cream variety. The nodule isolates from the brown variety are even poorly related to inoculants. This suggests poor competitive nodulation ability of our selected reference strains for the brown variety and strong inter-varietal differences in the choice of symbionts.

From the results emerging from the pot experiment, it can be concluded that the BGN varieties respond differently to different inoculant treatments. Even strain 9-5 previously preselected on the basis of plant performance and competition behavior on a local, undefined landrace of Bambara groundnut behaved quite differently when red, brown, or cream varieties of BGN were used instead. In addition, the indigenous strain(s) present in Kavango soil appeared to behave differently in terms of root nodulation according to the bean variety used. Therefore, to select an elite bioinoculant for three individual BGN varieties, the application of the seven individual reference strains separately and not as a mixture is required.

These varietal differences were also reflected in competition experiments using well-controlled, aseptic conditions in the phytotron, where from the mixture of seven strains, diverging isolates were the best competitors for the three Namibian cultivars. For the red variety, highly competitive strain C (9-5) also turned out to be the best for enhancing agronomic traits related to plant performance like plant height, leaf SPAD value, and shoot dry weight in single inoculation. On the other hand, each of the strains G (60 2-1) and A (1-7) induced the highest number of nodules in the red variety; the latter also expressed the second highest root nodule nitrogenase activity, which is comparable to that obtained with single inoculation with C (9-5). Highly competitive strain E (36 3-2) for the brown variety also exhibited the highest nodule nitrogen fixation on individual inoculation with this variety, whereas individual inoculant B (3B 4-1) elicited the highest SPAD-value and shoot dry weight measurements. In contrast, single inoculation with strongly competitive strain E (36 3-2) exhibited poor plant performance with the cream variety. On the other hand, strain A (1-7), the next best competitive strain for the cream variety, elicited a better plant performance on single inoculation. This stresses again that inoculant development for BGN is not an easy task, as bacterial strain–plant variety combinations seem to be quite specific.

Varietal differences in the response of BGN to different inoculants were also evident from single-inoculation experiments under aseptic conditions. In summary, selecting the best inoculant for red BGN is a challenging task. Strain C (9-5) showed the greatest improvement in plant growth and a comparatively high nitrogenase activity and is therefore probably the most promising candidate. Since strains F (55 1-1) and A (1-7) showed the highest and second highest nitrogenase activities, with A (1-7) inducing the highest mean nodulation, they could be further investigated as potential biofertilizer candidates for the red variety for field trials. On the other hand, D (36 1-1) treatment with no noticeable plant growth improvement together with the lowest nitrogenase activity is not advisable to choose as a bioinoculant for this variety.

Decision-making for selecting the best-performing inoculant exclusively for the brown variety is quite difficult as none performed consistently well for all the agronomic traits tested. Moreover, in most of the cases, the difference between inoculant treatment to untreated control was not very drastic. Strain B (3B 4-1) as well as A (1-7) performed consistently well for some of the parameters like plant height, chlorophyll content, and plant biomass, whereas E (36 3-2) and A (1-7) treatment, but not B (3B 4-1) treatment, performed relatively better with nodule nitrogenase activity, with E (36 3-2) even enhancing the nodule number. Therefore, it is difficult to choose a single strain as the best performer. However, based on relative overall performance strains A (1-7), B (3B 4-1), and E (36 3-2) have priority which could be confirmed from field performance on brown variety. In contrast, strain B (3B 4-1) performed relatively poorly on the red variety. Therefore, the varieties indeed respond differently to individual inoculants which once more justified that treating each variety with individual inoculants separately and not as a mix in field trials could be the right decision.

For the cream variety, the results substantiated from single plant data show good chances for three of the investigated rhizobia to bring agricultural benefits. Hence for the future, inoculation of the cream variety with B (3B 4-1), A (1-7), or C (9-5), respectively, might be interesting.

However, agronomic traits like plant shoot weight were not dramatically affected by inoculation. This is probably due to the large seed size of BGN providing nitrogen which will not be completely used up during our short incubation time of 6-8 weeks. Moreover, seed mineral concentrations of Bambara landraces have a significant impact on the early establishment of BGN; the dark-colored landraces have the highest concentration of macro and microelements compared to light-colored ones (Mandizvo and Odindo, 2019).

An unexpected visual observation from single-strain inoculation was the occurrence of dark-colored root nodules as a result of inoculation with strain 55 1-1(F) in all three varieties. Although very rare in occurrence, it was reported that *Rhizobium*-specific anthocyanin-like red pigments present in the nodule cortex of *Lupinus arboreus* were probably responsible for deep red nodule coloration (Caradus and Silvester, 1979). Later, during a program of screening rhizobia in West Africa, it was found that some strains induced nodules of unusually dark appearance on cowpeas, but not on other local beans tested. However, the dark pigmentation was in the bacteroid zone, not correlated with nodule effectiveness, and was additional to the leghemoglobin pigmentation (Eaglesham et al., 1982). Recently it was shown (Saad et al., 2019) that a deletion in *nifQ* of *Sinorhizobium (Ensifer) fredii* NGR234 (strain NGRΔnifQ) generated dark nodules of *Vigna unguiculata, V. radiata, and Macroptilium atropurpureum*. This peculiar dark-nodule phenotype coincided with a 20-fold or more accumulation of coproporphyrin III and uroporphyrin III in *V. unguiculata* nodules. Although nodule metal homeostasis was altered, it did not prevent the assembly and functioning of nitrogenase.

To develop an inoculant for the brown variety, nodule occupants that we detected in the uninoculated pot experiments may be instrumental. These indigenous rhizobias of Kavango soil nodulated this variety and appear to represent a novel cluster in the genus *Bradyrhizobium* according to phylogenetic analysis of the ITS sequences. The novel clade clusters with high bootstrap values and is not closely related to any type of strain and might harbor strains of a yet undefined species. It is generally believed that many African soils contain a diverse group of indigenous populations of *Bradyrhizobium* spp. that can nodulate and fix atmospheric nitrogen in several legumes (Ibny et al., 2019; Ajayi et al., 2020).

## 5 Conclusions

Bambara groundnut is an emerging yet neglected crop that has high potential to contribute to food security in Africa, especially due to adaptation to low-fertility soils and drought. To fully use its capacity for nitrogen fixation and thus thrive at low fertilizer doses in smallholder agriculture, a rhizobial inoculant should be developed. For Northern Namibia, our study started to develop bradyrhizobial inoculants adapted to three local BGN varieties and local soils. Our experiments using seven selected, indigenous temperature-resistant strains to test plant responses and competitiveness in soil and artificial substrates comprised important preparative tests to select strains for field experiments. Severe differences in symbiont-plant interactions appeared to occur in BGN depending on the plant variety, suggesting extensive screening is required to identify symbionts as inoculants that are sufficiently competitive and effective for certain plant genotypes. Our studies suggest there is a demand for coupling of breeding efforts with selecting efficient inoculant strains.

## Data availability statement

The original contributions presented in the study are included in the article/**Supplementary Material**. Further inquiries can be directed to the corresponding author. The DNA sequences obtained in this study were submitted to GenBank, GenBank accession numbers are OR392289 to OR392309.

## Author contributions

BR: Conceptualization, Funding acquisition, Investigation, Methodology, Project administration, Supervision, Writing – review & editing. AS: Conceptualization, Data curation, Formal Analysis, Investigation, Methodology, Supervision, Writing – original draft. FF: Data curation, Formal Analysis, Investigation, Methodology, Writing – original draft. SW: Formal Analysis, Investigation, Methodology, Writing – original draft. MD: Formal Analysis, Investigation, Writing – original draft. LK: Formal Analysis, Investigation, Writing – original draft. MZ: Formal Analysis, Investigation, Writing – original draft. LH: Conceptualization, Investigation, Supervision, Writing – review & editing.
