# Supplementary material for: Towards inoculant development for Bambara groundnut (Vigna subterranean (L.) Verdc) pulse crop production in Namibia
Source: Front Plant Sci. 2023 Oct 26;14:1270356. doi: 10.3389/fpls.2023.1270356 (PMC10641001; doi:10.3389/fpls.2023.1270356)
Supplement: Supplementary file 1 [file DataSheet_1.pdf]

frontiers

Supplementary Material

Supplementary Tables

Table S1 The mean-square values and significance tests of three varieties of Bambara groundnut treated with *Bradyrhizobium* strains as bio-inoculant evaluated in pots under greenhouse condition.

**Source of variation**	**df**	**DTE**	**PHT**		**DTF**	**Nodules per plant**	**SFW**	**SDW**
			**30 DAP**	**60 DAP**				
Replication	2	0.59	7.56	15.38	3.37	43.37	0.05	0.00
Variety	2	21.81***	13.54 ns	46.70 **	59.70 ***	9.93 ns	2.44 ***	0.09 ***
Inoculant	2	9.04 **	6.57 ns	2.13 ns	9.59 *	170.48 **	26.09 ***	0.72 ***
Variety x Inoculant	4	0.93 ns	1.80 ns	7.74 ns	6.26 ns	15.54 ns	1.13 ***	0.07 ***
Residual	16	0.84	4.31	5.22	2.66	18.45	0.54	0.00

Abbreviations: d.f.= degrees of freedom; DTE=Days to emergence; PHT= plant height; DTF, Days to flowering; Nodule_plant, Number of nodules per plant; SFW, Shoot fresh weight; SDW, Shoot dry weight; *, ** and *** denote significant at 5%, 1% and 0.1% probability level, respectively; ns = non-significant

Table S2 Pair-wise phenotypic correlation coefficients of agro-morphological traits among the Bambara groundnut (BGN) varieties treated with *Bradyrhizobium* strains as inoculant evaluated in pots under greenhouse conditions.

Variables	DTE	PHT 30 DAP	PHT 60 DAP	DTF	Nodule per plant	SFW	SDW
**DTE**	1	0.539499	0.52384	-.737*	-0.1941	0.194951	0.15469
**PHT-30DAP**	0.539499	1	.895**	-.730*	0.312151	.726*	.796*
**PHT-60DAP**	0.52384	.895**	1	-.700*	0.215294	0.421118	0.581084
**DTF**	-.737*	-.730*	-.700*	1	-0.32073	-0.44385	-0.51565
**Nodules / plant**	-0.1941	0.312151	0.215294	-0.32073	1	0.547754	0.60212
**SFW**	0.194951	.726*	0.421118	-0.44385	0.547754	1	.918**
**SDW**	0.15469	.796*	0.581084	-0.51565	0.60212	.918**	1

Abbreviations: d.f.= degrees of freedom; DTE=Days to emergence; PHT= plant height; DTF, Days to flowering; Nodules / plant, Number of nodules per plant; SFW, Shoot fresh weight; SDW, Shoot dry weight; *Correlation is significant at the 0.05 level (2-tailed). **. Correlation is significant at the 0.01 level (2-tailed).

Supplementary Figures

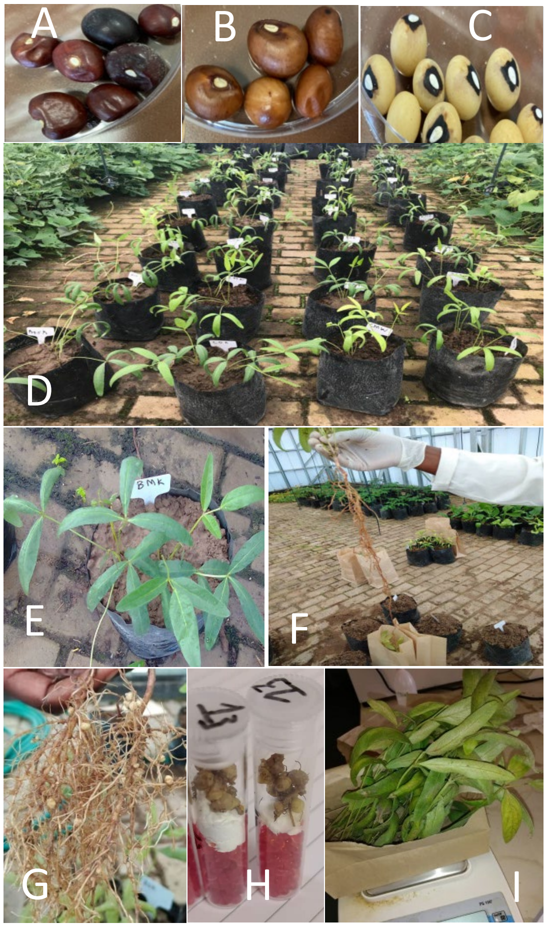

Figure S1. Bambara groundnut (BGN) pot experiments using Kavango soil in green house at UNAM-ZERI (Namibia) and the harvest. Three BGN varieties used in this study (A) red, (B) brown, and (C) cream, respectively (D) Set up of the pot experiments with three varieties of BGN involving three treatments (0K, no inoculation; 9-5K, single inoculant strain 9-5; MK, mixed inoculant with seven strains of *Bradyrhizobium* spp.) in several replicates. (E) A representative photo showing one BGN-plant with label BMK (brown variety in Kavango soil inoculated with MK) which is ready for harvest after 60 days of inoculation. (F) BGN plant carefully uprooted from the soil with hand so that the shoot and the root system remain intact (G) Harvested root with inoculant treatment having plenty of root nodules (H) Collected root nodules being stored in dry silica gel vials for transport and future analysis (I) Weighing of the above ground parts of BGN after harvest.

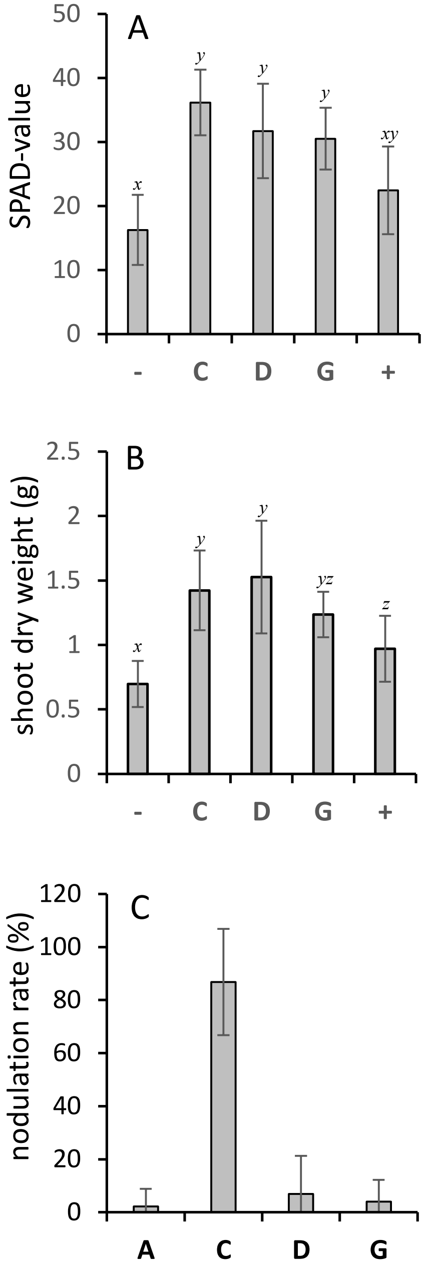

Figure S2. Effect of single strain inoculation with four of the seven reference strains on plant growth parameters as well as competing nodule occupancy on a Namibian local undefined landrace of Bambara groundnut under aseptic conditions in the phytotron. Incubated for 6-7 weeks after inoculation. Results represented as bar diagrams with standard errors; -, negative control without inoculant, no nitrogen; +, positive control with 5 mM potassium nitrate as N source; strain designation **A**, 1-7; **C**, 9-5; **D**, 36 1-1; **G**, 60 2-1. (A) mean SPAD value of the leaves of the plants; (B) mean shoot dry weight of the plants; (C) competitiveness for nodule occupancy after inoculation with the mixed inoculant (in equal proportion of each of the four reference strains). The mean change factor in each case was converted to percentage of each of the strains of the mixture. The details of calculation are described in Materials and Methods. All data were collected from three independent biological replicates (each with 2-3 technical replicate plants). Treatments with single reference strains labeled with different letters in (A) and (B) indicate statistical significance ($p<0.05$) calculated using one-way ANOVA.

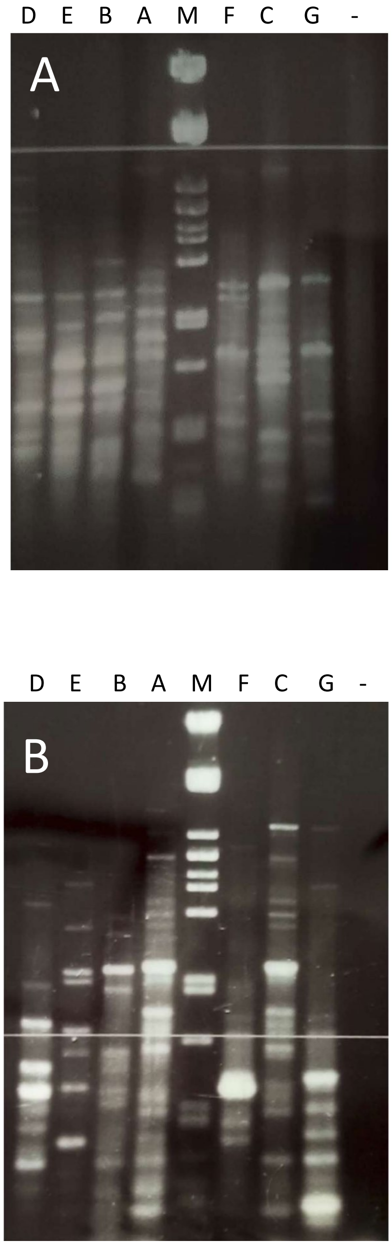

Figure S3. Rep-PCR mediated genomic fingerprinting to distinguish between strains of *Bradyrhizobium* in a mixed inoculant for BGN inoculation. Agarose gel electrophoretic representation of (A) BOX-PCR and (B) ERIC-PCR based DNA finger-printing banding pattern from pure cultures of the seven individual strains. In each case 7 µl of PCR product was loaded in each lane from a 50 µl PCR-reaction in 1.5% Agarose gel in 1X TAE buffer and stained later with ethidium bromide. DNA-fragments of variable lengths generated distinct "fingerprint" for each strain. For (A) and (B) order of loading in the individual lanes from left to right: D: 36 1-1; E: 36 3-2; B: 3B 4-1; A: 1-7; M: DNA size marker (λ-DNA digested by *Pst*I); F: 55 1-1; C: 9-5; G: 60 2-1; negative control, no template.
